# A quasi-experimental study on health insurance coverage and health services in Nigeria

**DOI:** 10.4102/phcfm.v16i1.4056

**Published:** 2024-01-21

**Authors:** Shailender Singh, Meenakshi Kaul, Muhammad M. Bala, Chitra Krishnan, Chandrashekhar J. Rawandale

**Affiliations:** 1 Symbiosis Centre for Management Studies, Symbiosis International Deemed University, Noida, India; 2 Symbiosis Law School, Symbiosis International Deemed University, Pune, India; 3 Department of Economics, School of Liberal Arts and Social Sciences, SRM University-AP, Amaravati, India

**Keywords:** health insurance coverage, care utilisation, facility delivery, public facilities, private facilities

## Abstract

**Background:**

Nigeria has the highest maternal mortality rate among sub-Saharan African countries. Recently, universal health insurance coverage has been embraced as a means to enhance population health in low- and middle-income countries. Hitherto, the effect of health insurance coverage on the utilisation of facility-level delivery is largely unknown in the face of the earnest need to lower maternal mortality rates in developing countries.

**Aim:**

To empirically investigate the association of health insurance coverage on health services utilisation of facility-level delivery and the extent to which public- and private-sector facility delivery in Nigeria had a disproportionate associational effect with health insurance coverage, in the universal health coverage era.

**Setting:**

A cross-sectional study conducted for Nigeria.

**Methods:**

This study employed a quasi-experimental method using propensity scores along with different matching methods that were applied to the most recent wave of Nigeria’s Demographic and Health Survey (2020) data.

**Results:**

Evidence suggests that childbearing mothers from insured households had an average of 25% probability of utilising facility-level delivery relative to mothers from uninsured households in the year that preceded the survey. Moreover, private-sector facility delivery had a 31% higher associational effect with health insurance coverage than public-sector facility delivery, which had an estimated probability of 21%.

**Conclusion:**

Expansion of health insurance coverage in Nigeria will be a desirable way to stimulate the utilisation of facility-level delivery by women of childbearing age. Consequently, coverage expansion has the potential to save many maternal and newborn lives in Nigeria.

**Contribution:**

This study has contributed to the urgent attention of the federal government of Nigeria to monitor and revamp the health insurance coverage policies of the country for better facilitation of health services to the Nigerian population.

## Introduction

The Sustainable Development Goals (SDG) target 3.1 aims to lower maternal maternity rates globally to below 70 per 100 000 live births by 2030 compared to 2015 figures.^1^ Reducing maternal mortality rates remains a global priority to date. Nigeria is one of the low- and middle-income countries where progress in achieving SDG 3.1 has been quite low. According to 2017 UNICEF estimates, Nigeria has a maternal mortality rate of 917 per 100 000 live births.^2^ Thus, Nigeria’s estimates are among the highest globally. However, many of these deaths could be prevented in a setting with a strong well-equipped healthcare system handled by skilled birth attendants. Therefore, expanding access to facility-level delivery will be a powerful way to increase utilisation of facility-based delivery and in turn lower maternal mortality rates.^3^

Accordingly, the World Health Organization (WHO) and other key development partners, such as the World Bank, have repeatedly called on countries to improve population health and reduce household exposure to vulnerability by embracing universal health coverage (UHC). In its simplest form, UHC is attained when all people are provided with a wider range of health services they need without imposing financial hardship or leading to household impoverishment from catastrophic out-of-pocket expenditures.^4^ It generally targets the expansion of health insurance coverage to everyone by providing good health services – ranging from preventive to promotive, treatment, and palliatives which ensures financial risk protection.^5^

Prima facie economic theory asserts that health insurance coverage induces greater utilisation of health services, reduces household exposure to financial hardships, improves population health and reduces morbidity and mortality, regardless of the existing healthcare system in a given context.^6^ However, available evidence, particularly from low- and middle-income countries, suggests that expansion of health insurance coverage augmented demand for routine care such as maternal and child health; hitherto, there is a dearth of evidence on the effects of health insurance coverage on the facility-level delivery in many resource-constraint countries.

Many studies have provided different evidence for the effect of health insurance coverage on care utilisation, financial risk protection, better self-reported health and lowered population morbidity and mortality rates, especially in high-income countries. In many low- and middle-income countries, studies have documented substantial evidence that health insurance coverage generally increases the demand for medical services. However, evidence on better financial risk protection and mortality reductions has been conflictual. For instance, the implementation of an extensive programme termed *Janani Suraksha* in India in the past 15 years, which offered cash inducements for women to deliver their babies in hospital facilities, increased facility-level birth by over 50 million women in the country; there is no significant evidence on reductions in maternal and newborn survivals, nonetheless.^7^ In the Philippines, Gouda and colleagues^8^ found that insured women had a 5% – 10% higher probability of facility-level delivery than the uninsured. Health insurance coverage is significantly associated with using different types of maternal health services but does not affect facility-level deliveries in Ghana.^9^ In Indonesia, health insurance status, among other key factors, is associated with facility-level deliveries attended by skilled birth attendants.^10^ A systematic review of quantitative literature that examines the effect of health insurance coverage on the utilisation of maternal and neonatal health services shows positive results.^11^ A comparative study between Ghana, Indonesia and Rwanda concludes that health insurance uptake significantly affects maternal healthcare utilisation in at least two of the four measures considered in the study.^12^ In Kenya, institutional delivery is more likely associated with insured mothers compared to uninsured mothers.^13^ Antenatal care and facility base deliveries had a statistically significant impact on insured wealthy households than those without coverage in Gabon.^14^

Therefore, this is the first study investigating the effects of health insurance coverage on care utilisation of facility-level delivery and the extent to which public- and private-sector facility-level delivery disproportionately affects health insurance coverage in Nigeria.

## Research methods and design

This study uses the most recent wave of a cross-sectional nationally representative household-level Demographic Health Survey (2020) data of Nigeria conducted by the National Population Commission (NPC). The data were collected in 2020 across the 36 states of Nigeria, including the Federal Capital Territory using stratified two-stage cluster random sampling to ensure national representation of the enumeration units – the clusters. In each cluster, 30 households were randomly selected for the survey, and the respondents were either the head of the household or any adult responsible for implementing major household decisions. Overall, 40 427 households were surveyed, comprising 41 821 women between the ages of 15 years and 49 years and 13 311 men within the ages of 15 years – 59 years, respectively.

More precisely, information on health insurance coverage and healthcare utilisation of facility-level delivery was obtained from the household core questionnaire and the women’s questionnaire. The survey asked household respondents the question: ‘Are you covered by health insurance?’ Only 739 respondents representing 3.25% of the population had health insurance coverage at the time of the survey. Similarly, regarding the health outcome, the survey asked mothers the question: ‘Where did you give birth to (name of the child)?’ The mother responding to the question had several options to choose from the list, including government hospitals, government health centres, private hospitals, private clinics and homes, among others. Accordingly, 6309 women representing 70.09% of the population had public-sector facility-based deliveries, and 2692 representing 29.9% women had private-sector child deliveries 12 months before the survey period. This study focused on facility-level delivery by women, primarily for the fact that women due for child delivery are in need of specialist care at the facility for quick identification of any possible complication that may cost the lives of either the mother or the child or both. Also, facility-level delivery by women represents the health system’s readiness to provide maternal care with a skilled birth attendant and the women’s confidence to utilise such services whenever they are due for child delivery.^8^

### Estimation method

This study applied the propensity score matching technique to empirically examine the effects of health insurance coverage on health services utilisation of facility-level delivery by women of childbearing age. Other matching methods were also applied to check the robustness of the results with different estimation methods. These analytical techniques are mostly used to arrive at unbiased estimates of the effect of a particular treatment on the outcome of interest. However, given that people may not randomly buy health insurance schemes, this study estimates causal inference using quasi-experimental methods in line with the existing literature.^8^ Propensity score matching, a quasi-experimental method, is best suited for studies constructing a control group by matching each treated unit with a non-treated unit. In the present study, individuals with health insurance coverage are regarded as the treated group, and those without health insurance coverage are considered as the non-treated group. A large pool of observable characteristics is maximised to obtain the control observations that are closely similar to the treated units.^15^ In an observational study using cross-sectional data, information is only available for individuals who are either insured or not. Rosenbaum^16^ has suggested the application of balancing scores to match insured and non-insured individuals based on individuals who share similar attributes. The matching is predicated upon individual observable attributes that are independent of the insurance status.^17^

The effect of insurance coverage on an outcome is the average treatment effect on the treated (ATT). This implies that there is a ‘region of common support’ (i.e. there is a fair probability of obtaining health insurance coverage for both the treatment and the control group over the span of approximated propensity scores). Thus, the propensity score obliterates selection bias because of observable covariates, which satisfies the conditional independence assumption.^16^

$$ATT=E[\tau |M=1]=E[{\text{Y}}_{1}|D=1]-E[{\text{Y}}_{0}|M=1]$$ [Eqn 1]

where M є {0,1} is a binary indicator that indicates the treatment of health insurance coverage and represents the potential utilisation of health services for both the treated and the control groups, X indicates a vector of covariates, and E denotes the mean operator of the population. Moreover, the estimation of the ATT parameters demands that each treatment effect is unconnected with the treatment status of other individuals. Heckman^18^ and colleagues have suggested that the premise of mean independence of potential outcome given a set of covariates X and the overlap condition are enough to express the ATT in Equation 2 and Equation 3 using a matching method:

$$E[{\text{Y}}_{1}|D=1,X]=E[{\text{Y}}_{1}|M=0,X]=E[{\text{Y}}_{1}|X]$$ [Eqn 2]

P(D=1|X=x)<1 [Eqn 3]

The propensity scores are computed using a probit model. Notably, many variables influence an individual decision to obtain health insurance coverage and potential utilisation of care that are not affected by the coverage.^18^ Thus, the pertinent household characteristics that affect health insurance coverage included in the probit model are age, education, employment and income level.

### Ethical considerations

This article followed all ethical standards for research without direct contact with human or animal subjects.

## Results

This section begins with the analysis of the probit regression model. Table 1 illustrates descriptive statistics and two-sided tests for equality of facility-level delivery by mothers in the past 12 months that preceded the survey by health insurance coverage status. The mean health services utilisation shows that the share of women who had facility delivery does not significantly differ by insurance status, to begin with. Moreover, the exact opposite holds true with health services utilisation between public- and private-sector delivery and health insurance coverage. The mean facility-level utilisation is higher for private-sector delivery relative to the public sector, which is not surprising because of the difference in the quality of services rendered by private-sector providers in the country.

**TABLE 1:** Descriptive statistics of facility-level delivery by insurance status.

Child delivery	With health insurance coverage Mean	Without health insurance coverage Mean	Test for equality (insured vs. uninsured) *z*-statistics
Facility-based delivery	24.4033	24.4199	0.8383
Public-sector delivery	21.2723	21.5879	13.1123**
Private-sector delivery	31.0431	31.0854	1.2045*

* , significant at 5%;

** , significant at 1% levels.

Table 2 shows the descriptive statistics and the two-sided test between the set of covariates included in the model and health insurance coverage status. The set of covariates considered are variables that are intuitively expected to influence the probability of obtaining health insurance coverage and utilisation of health services. Therefore, in line with underlying economic theory and empirical evidence, securing health insurance coverage in Nigeria is a rare event in the population. The distribution of health insurance is more common among federal government workers through the mandatory National Health Insurance Scheme; hitherto, many state governments do not provide mandatory health insurance coverage to their workers.

**TABLE 2:** Descriptive statistics of the model covariates by insurance status.

Socio-demographic covariates	With health insurance coverage Mean	Without health insurance coverage Mean	Test for equality (insured vs. uninsured) *z*-statistics
Age of household age	47.7780	48.8829	84.4049***
Occupation	3.2694	5.1138	118.8461***
Educational attainment	4.4460	2.7401	−98.6228***
Sex of household age	1.2449	1.2376	−0.5629
Wealth index	4.5602	3.3077	−95.6806***
Marital status	0.7469	0.7636	1.2735*
Region	3.4343	3.4253	−0.6858

* , statistically significant at 10% levels;

** , statistically significant at 5% level;

*** , statistically significant at 1% level.

Moreover, the age of the household head, occupation, sex and marital status have been included because of their vital role at the household level in deciding to obtain health insurance coverage. Also, the wealth index is included among the set of covariates in order to account for the differences in the household’s ability to obtain health insurance coverage, which significantly affects demand for health and healthcare utilisation. The inclusion of the region is meant to capture geographical differences in access to care in Nigeria. Based on these covariates, it is seen that households with health insurance coverage differ significantly from those who do not have insurance coverage. The statistics mostly supported the notion that obtaining coverage is strongly correlated with the set of covariates included.

Table 3 presents the probit model regression estimates of the factors that may predict an individual’s health insurance status. Although the age of the head of the household, educational attainment, household occupation and household wealth index significantly predict health insurance status, the sex of the household head is not a statistically significant determinant of health insurance coverage after several covariates were controlled. Moreover, from the statistically significant predictors for health insurance coverage, the household wealth index had the weightiest effect on health insurance coverage, followed by their level of education. Household occupation, though significant, bears a negative sign. This may be connected to the existing reality that formal sector employment as a share of the total labour force in the country is relatively low. Thus, obtaining health insurance coverage is more likely for wealthier and more educated households in Nigeria. Additionally, based on pseudo-*R*^2^, chi-square tests and log-likelihood ratio, the null hypothesis is rejected.^17^

**TABLE 3:** Probit regression model estimates.

Covariates	Coefficient	Std. error	*z*	*P > z*	95% CI
Age of the head of household	0.0070*	0.002	2.79	0.005	0.00–0.01
Occupation	−0.0603*	0.018	−3.41	0.001	−0.09–0.02
Sex	0.0173	0.087	0.20	0.843	−0.15–0.18
Education	0.3236*	0.038	8.46	0.000	0.24–0.39
Wealth index	0.3673*	0.044	8.30	0.000	0.28–0.45
Constant	−4.6322*	0.301	−18.39	0.000	−5.22–4.04

Note: Log-likelihood = -963.46; Pseudo -*R*^2^ = 0.19; Wald test *χ*^2^ = 454.72***; Number of observations = 7219; Probability 0.01.

* , implied statistically significant at 1%.

Table 4 presents the estimates of the associational effect of health insurance coverage on health services utilisation of facility-level delivery in Nigeria. Overall, the estimate supports the underlying notion of economic theory that health insurance coverage has a statistically significant impact on the utilisation of health services in both the public and private sectors. It stands out that mothers from households covered by health insurance had an average of 25% chance to access facility-level delivery in a year that preceded the survey. Also, those with health insurance coverage had a 31% higher probability of accessing private-sector facility delivery than public-sector facility delivery, which had an estimated probability of 21%. The estimates are statistically significant across alternative matching techniques.

**TABLE 4:** Effects of the health insurance coverage on facility-level delivery of child in Nigeria.

Child delivery	Propensity score matching		Nearest neighbour matching		Kernel matching	
	ATT	s.e.	ATT	s.e.	ATT	s.e.
Facility-based delivery	0.2458*	0.464	0.2458*	0.464	0.2458*	0.464
Public-sector delivery	0.2130*	0.065	0.2130*	0.065	0.2130*	0.065
Private-sector delivery	0.3105*	0.163	0.3105*	0.163	0.3105*	0.163

ATT, average treatment effect on the treated; s.e., standard error.

* , implied statistically significant at 10% level.

**FIGURE 1:**
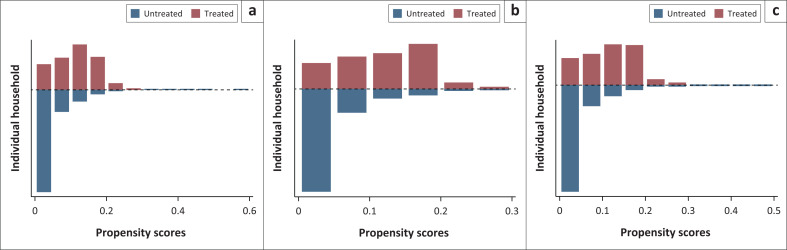
Propensity scores graph of (a) facility based delivery, (b) public sector delivery and (c) private sector delivery.

Furthermore, this analysis evaluates the quality of the different matching techniques concerning the underlying assumption of conditional mean independence. Figure 1 depicts the propensity score graphs for the treated and untreated coverage effects on health services utilisation under investigation. There are high levels of common support between the treated and the control group because the numbers of observations that are off support are quite small. Individual household members in the blue represent the untreated, and the one in the red represents the treated. Thus, their evidence of overlapping propensity scores is clear evidence of common support, as earlier indicated by the propensity score output estimates. In addition to graphic representation checks, this analysis further evaluates the propensity scores match using the propensity score test, and the result indicates that the estimates have achieved some balance between the observable covariates.

## Discussion

This study examines the effects of health insurance coverage on health services utilisation, specifically for facility-level delivery among women of childbearing age in Nigeria. Noteworthy, this study finding is consistent with the crux of economic theory that health insurance coverage is associated with greater utilisation of health services for a wider range of conditions in a given context, regardless of the nature of the health system. The primary outcome suggests that mothers from insured households have an estimated 25% probability of utilising facility-level delivery in Nigeria. Also, those with health insurance coverage had a 31% higher probability of utilising private-sector facility delivery than public-sector facility delivery, which had an estimated probability of 21%.

Therefore, these findings are consistent with what was earlier reported by Powell and colleagues in an Indian context, even though this study was limited to the examination of coverage effects on utilisation of facility-level delivery without extending it to the examination of coverage effect on maternal and newborn survivals, as it was the case in their study.^7^ It is also incongruent with the evidence documented in the Philippines by Gouda et al.^8^ and that of Nasution et al.^10^ where coverage was among other factors associated with facility-level delivery with a skilled birth attendant in Indonesia. In contrast, this result contradicts the findings of a study in Ghana, where coverage stimulates the utilisation of maternal care but is not associated with facility-level delivery.^9^

Notably, the findings align with what the promoters of UHC–WHO and the World Bank have been advocating for. Though in 2017, health expenditure as a total share of GDP was only 3.8% and health expenditure per capita was estimated at 74 USD in Nigeria, these figures are by and large considerably low relative to the global efforts to curtail maternal and neonatal mortality rates in a country which accounted for 20% of global maternal mortality rates.^19^ Despite the remarkable decline in maternal death in Nigeria from 1200 to 917 women per 100 000 live births between 2000 and 2017, the pace of the decline is far below the level required to attain SDG 3.1 by 2030.

Therefore, this study suggests a massive expansion of health insurance coverage to incorporate the informal sector, which accounts for the largest segment of the population in both urban and rural areas. Similarly, the state government should also introduce mandatory health insurance coverage to their entire workforce, similar to what is being provided by the federal government of Nigeria. In the same way, coverage expansion will only produce the desired results when the supply side is also enhanced for efficient service delivery. The government should also increase its health expenditure as a total share of GDP so that needed investment can be made into the high-quality health system that provides a broader range of services for maternal and child health and other conditions with high mortality and morbidity burden in the country.

## Conclusion

Mothers from insured households had a 25% probability of utilising facility-level delivery relative to uninsured mothers prior to the most recent wave of the DHS survey in Nigeria. In addition, private-sector facility delivery has 31% higher associational effects with health insurance coverage compared to public-sector facility delivery, with an estimated probability of 21%. This evidence suggests that health insurance coverage exerts more impact on facility-level delivery in Nigeria. Therefore, coverage expansion may automatically trigger greater utilisation of facility-level delivery, which has the potential to save many maternal and neonatal lives in the country, in spite of the existing low-quality healthcare system in Nigeria.

Therefore, because this study established evidence that health insurance coverage is associated with the utilisation of facility-level delivery, providing further empirical evidence to the effect of health insurance coverage on maternal and neonatal survival in the Nigerian context will be of immense policy implications. This research work is not without some limitations. In quasi-experimental studies, groups may differ systematically in several ways at baseline, and these differences will influence the outcome of interest, which may generate misleading results. Multivariable regression is therefore a better approach of data analysis, because the effects of confounding variables could be adjusted for in multivariable regression.
